# Folate hydrolase-1 (FOLH1) is a novel target for antibody-based brachytherapy in Merkel cell carcinoma

**DOI:** 10.1002/ski2.9

**Published:** 2020-11-28

**Authors:** M. K. Ramirez-Fort, B. Meier-Schiesser, K. Lachance, S. S. Mahase, C. D. Church, M. J. Niaz, H. Liu, V. Navarro, A. Nikolopoulou, D. V. Kazakov, E. Contassot, D. P. Nguyen, J. Sach, L. Hadravsky, Y. Sheng, S. T. Tagawa, X. Wu, C. S. Lange, L. E. French, P. T. Nghiem, N. H. Bander

**Affiliations:** 1 Department of Life Sciences, BioFort®, Guaynabo, Puerto Rico, USA; 2 Department of Urology, Weill Cornell Medicine, New York, New York, USA; 3 Department of Radiation Oncology, SUNY Downstate Health Sciences University, Brooklyn, New York, USA; 4 Department of Dermatology, University Hospital of Zürich, Zürich, Switzerland; 5 Department of Dermatology, University of Washington, Seattle, Washington, USA; 6 Department of Radiation Oncology, Weill Cornell Medicine, New York, New York, USA; 7 Department of Radiology, Weill Cornell Medicine, New York, New York, USA; 8 Sikl’s Department of Pathology, Medical Faculty in Pilsen, Charles University in Prague, Pilsen, Czech Republic; 9 Shanghai Proton and Heavy Ion Center, Shanghai, China; 10 Department of Medicine, Weill Cornell Medicine, New York, New York, USA; 11 Innovative Cancer Institute, Miami, Florida, USA; 12 Department of Dermatology, Münich University Hospital, Münich, Germany

## Abstract

**Backgrounds:**

Folate Hydrolase-1 (FOLH1; PSMA) is a type II transmembrane protein, luminally expressed by solid tumour neo-vasculature. Monoclonal antibody (mAb), J591, is a vehicle for mAb-based brachytherapy in FOLH1+ cancers. Brachytherapy is a form of radiotherapy that involves placing a radioactive material a short distance from the target tissue (e.g., on the skin or internally); brachytherapy is commonly accomplished with the use of catheters, needles, metal seeds and antibody or small peptide conjugates. Herein, FOLH1 expression in primary (p) and metastatic (m) Merkel cell carcinoma (MCC) is characterized to determine its targeting potential for J591-brachytherapy.

**Materials & Methods:**

Paraffin sections from pMCC and mMCC were evaluated by immunohistochemistry for FOLH1. Monte Carlo simulation was performed using the physical properties of conjugated radioisotope lutetium-177. Kaplan–Meier survival curves were calculated based on patient outcome data and FOLH1 expression.

**Results:**

Eighty-one MCC tumours were evaluated. 67% (54/81) of all cases, 77% (24/31) pMCC and 60% (30/50) mMCC tumours were FOLH1+. Monte Carlo simulation showed highly localized ionizing tracks of electrons emitted from the targeted neo-vessel. 42% (34/81) of patients with FOLH1+/− MCC had available survival data f or analysis. No significant differences in our limited data set were detected based on FOLH1 status (*p* = 0.4718; *p* = 0.6470), staining intensity score (*p* = 0.6966; *p* = 0.9841) or by grouping staining intensity scores (− and + vs. ++, +++, +++) (*p* = 0.8022; *p* = 0.8496) for MCC-specific survival or recurrence free survival, respectively.

**Conclusions:**

We report the first evidence of prevalent FOLH1 expression within MCC-associated neo-vessels, in 60-77% of patients in a large MCC cohort. Given this data, and the need for alternatives to immune therapies it is appropriate to explore the safety and efficacy o f FOLH1-targeted brachytherapy for MCC.

The incidence rates of Merkel cell carcinoma (MCC), an aggressive cutaneous malignancy, have tripled from 0.15 cases per 100 000 individuals in 1986 to 0.7 per 100 000 in 2013, corresponding to 2488 cases/year.^1–3^ MCC is three times more lethal than melanoma, with a 46% disease-associated mortality rate, and 5-year disease-specific survival rates of 66% and 11%–30% for local and metastatic disease, respectively.^2,4^ These concerning survival rates reflect MCC’s propensity for local recurrence and regional nodal involvement,^5^ where 30-50% of locally staged patients eventually develop a distant metastasis.^4,6^

MCC is often diagnosed late in its pathogenesis, thereby requiring systemic approaches early in management. Systemic therapy using standard chemotherapeutic agents such as etoposide, epirubicin, doxorubicin, cyclophosphamide and cisplatin-based chemotherapeutic regimens as radiosensitizers or as definitive treatment remains disappointing. These systemic agents are associated with high toxicity rates, transient responses, resistance and no overall survival benefits.^7–10^ MCC’s poor prognosis, high recurrence and mortality rates, in parallel with disappointing outcomes associated to conventional treatments, warrant evaluating alternative therapeutic modalities. The need for novel strategies to treat rare diseases like MCC is recognized by the U. S. Food and Drug Administration (FDA), assisting in drug development for diseases affecting fewer than 200 000 people by providing financial and logistical incentives.

Recent studies showing PD-L1 expression in the tumour microenvironment of MCCs, and PD-1 expression by MCC-specific tumour infiltrating and circulating T cells, supported investigating the utility of immune checkpoint inhibitors.^11,12^ A phase 2 trial of avelumab (a human anti-PD-L1 IgG1 monoclonal antibody [mAb]) in patients with metastatic (m) MCC that was refractory to chemotherapy, demonstrated a 31.8% durable objective response rate, according to Response Evaluation Criteria in Solid Tumors version 1.1,^13,14^ culminating in avelumab being the first FDA-approved immunotherapy for MCC in March 2017.

Despite these promising results with use of targeted immunotherapy, approximately half of patients do not respond to PD-1/PD-L1 axis targeting, necessitating development of alternative or additional therapeutic strategies. MCC tumours and metastatic deposits have large mitotic fractions that are susceptible to ionizing radiation damage. They are highly vascularized, leading to intrinsic sensitivity to photon and electron external beam radiation therapy (EBRT) via an increased oxygen enhancement ratio. Several studies and the NCCN guidelines promote definitive or adjuvant EBRT to optimize localized tumour control in primary (p) MCC that are surgically inoperable, resections with borderline or positive margin status, tumours greater than 1 cm in the context of a positive lymph node biopsy, presence of lymphovascular invasion, and history of immunodeficiency.^15,16–20,21^

Although MCC is intrinsically radiosensitive, the field size of conventional treatment with EBRT for widespread disease is limited by the radiation tolerance of normal tissues or organs at risk surrounding tumour deposits (e.g., optic nerve, spinal cord, lung).

Brachytherapy is an alternative form of radiotherapy that involves placing a radioactive material a short distance from the target tissue (e.g., on the skin or internally); brachytherapy is commonly accomplished with the use of catheters, needles, metal seeds and antibody or small peptide conjugates. Antibody-based brachytherapy is a clinically validated form of radiation therapy that uses a mAb to deliver radioactive isotopes directly to sites of local and metastasized cancer.^22–29^ The intrinsic properties of radioisotope lutetium-177 (^177^Lu), make it an ideal candidate for antibody-based brachytherapy; ^177^Lu decays by β particle (i.e., electrons) emission (0.497 MeV; *t*_1/2_ = 6.74 days) of relatively short-range (0.2–0.3 mm). The calculated optimal tumour size for treatment with ^177^ Lu is thought to be 1.2–3 mm ^30^. For larger tumours, the clinical efficacy of longer range β emitters has been demonstrated, for example, yttrium-90 (^90^Y).^31^ Clinical data on FOLH1- targeting with α-emitters (i.e., two protons and two neutrons) in prostate cancer (PCa) is promising and suggests that α-emitters could be considered for hypoxic tumours.^32^ Notably, the micro-dosimetry of antibody or peptide-based brachytherapy is currently in development.

The mAb J591 has demonstrated excellent tumour theragnostic specificity in PCa, and in the neo-vasculature of several solid tumours, via targeting of membrane-expressed (FOLH1; also known as prostate-specific membrane antigen) FOLH1.^22,24,25,27,33^ FOLH1 is a transmembrane enzyme receptor that is upregulated on the cell membrane of PCa, intracellularly in melanoma cells and the neo-vascular lumen of virtually all solid tumours (including PCa and melanoma).^22,34^ Importantly, there has not been a single patient reported to have toxicity to non-tumour-related vessels.

*In vivo* targeting of FOLH1 by conjugating auristatin (a cytotoxic agent) to J591 increased the therapeutic index of auristatin by 700-fold, and improved the median survival of PCa xenografts by 9 months.^35^ Clinically, ^177^Lu-J591 is well-tolerated, non-immunogenic, and can be fractionated. Studies with unconjugated J591 (i.e., mAb only) report no dose-limiting toxicities in patients with solid tumours.^36^ An ongoing trial is investigating *in vivo* localization of metastasized solid tumours with FOLH1 PET/CT imaging using positron emitting zirconium-89 (^89^Zr)-J591, and the effects on tumour perfusion and cellularity with a cumulative dose of 70 mCi of ^177^Lu-J591 (NCT00967577).

Herein we aim to validate the expression of FOLH1 in pMCC and mMCC. Paraffin sections of pMCC and mMCC were provided by academic medical centers in Switzerland, Czech Republic, Germany and the United States. A total of 81 MCC tumours were evaluated for FOLH1 expression by standard immunohistochemistry. One pMCC and one mMCC tumour was obtained from the same patient. Primary antibodies used were 3E6 (DAKO) mouse IgG1 monoclonal anti-human FOLH1, mouse IgG1 isotype antibody (Abcam) and anti-CD31 (IgG1, Abcam). Mouse IgG1 isotype antibody (Abcam) at corresponding concentrations was used as a negative control. Anti-CD31 (IgG1) was used as a positive control to stain vasculature. Biotinylated goat-anti-mouse (Southern Biotech) was used as a secondary antibody. Sections were imaged with Aperio ScanScope (Leica Biosystems). The MCC cases were classified as (−), (+), (++), (+++) and (++++) staining intensities; (++++) was characterized as maximal staining as seen in PCa. Three dermatopathologists in New York, United States reviewed all slides for FOLH1 immunostaining and intensity. The slides were reviewed independently and together; in the setting of inter-observer measurement difference, the measurement with majority agreement was used.

Overall, 67% (54/81) of cases showed FOLH1^+^ neo-vessels. 77% (24/31) of pMCC cases and 60% (30/50) of mMCC cases demonstrated FOLH1-positivity. FOLH1^+^ was restricted to MCC neo-vessels (as confirmed by co-labelling with anti-CD31) (Figure 1a,b). No FOLH1 tumour cell staining was identified. The majority of FOLH1^+^ vessels were identified in the periphery of infiltrating tumour cells. One FOLH1^+^ paraffin section contained both p- and mMCC tumours (from the same patient), suggesting that biopsy of pMCC can predict some degree of FOLH1^+^ homogeneity in a meta-stasis. Semi-quantification of FOLH1 staining intensity showed that both pMCC and mMCC expressed significantly more FOLH1 as compared to healthy skin (Figure 2).

Monte Carlo simulation with MCNPX (V2.5.0) was used to calculate the tracks of 30 emitted electrons and ionization tracks in water media (as a serrogate for measuring absorbed dose in tissue or tumour) to show the penetration characteristics of ^177^Lu electrons (0.147 MeV mean energy^37^) from a single point. The total range of the electrons at such energy level is in the order of 200 μm. The electron ionization tracks originating from a single point in water were then superimposed onto a neo-vessel in the histopathological image of a FOLH1^+^ MCC tumour to demonstrate the algorithmic predicted dose deposition points generated by FOLH1 targeting of a single point along a neo-vessel, and in a single direction perpendicular to blood flow (Figure 3). As supported by our Monte Carlo simulation, ^177^Lu-J591 provides a highly localized dose distribution, which permits specific, systemic targeting of disseminated disease with ionizing irradiation while limiting irradiation beyond the tumour boundaries (Figure 3).

Statistical analyses on patient survival were performed using Stata software version 14.0 (StataCorp). Survival analyses were calculated from time of diagnosis to the outcome event. For MCC-specific survival, an event was defined as death by MCC. Recurrence-free survival’s event was either a MCC recurrence or death by MCC. Patients that were lost to follow-up were censored in all analyses. Kaplan–Meier survival curves were created to visualize patient survival outcome based on FOLH1 tumour expression (Figure 4). The log-rank test was performed to determine if there were differences between FOLH1 status groups and a *p* value less than 0.05 was considered statistically significant.

Patient demographics, survival outcome, and FOLH1 neo-vessel expression data was available and evaluated in a cohort of 35/81 MCC patients (Table 1). Patients with FOLH1^+^ (in each group of staining intensity) and FOLH1^−^ MCC were demographically homogeneous with regards to sex, age at diagnosis, immunosuppression status, prior therapies, stage at diagnosis and local or distant recurrences. No significant differences in our limited data set were detected based on FOLH1 status (*p =* 0.4718; *p =* 0.6470), staining intensity score (*p =* 0.6966; *p =* 0.9841), or by grouping staining intensity scores (− and + vs. ++, +++, +++) (*p =* 0.8022; *p =* 0.8496) for MCC-specific survival or recurrence free survival, respectively. Given the rarity of MCC, our results are not powered to show that there is no significant difference (Table 1).

Other studies have shown FOLH1 expression is not prognostic in renal cell carcinoma,^38^ but has prognostic implications in multiple other solid tumours.^39–42^ Nevertheless, MCC expression of FOLH1 is significantly more common than the observed expression of human epidermal growth factor receptor-2 (HER-2) in breast cancer (16%; 95% CI, 12%, 21%),^43^ and than the reported ∼49% PD-L1 and ∼55% PD-1 expression on MCC tumour cells and infiltrating lymphocytes, respectively.^12^ Comparison of FOLH1 target expression to HER-2, PD-L1 and PD-1 expression, suggests that FOLH1 may be a viable therapeutic target for MCC patients, particularly for treatment with antibody- or peptide-based brachytherapy.

As stated above, need for novel strategies to treat rare diseases like MCC is recognized by the US FDA. Targeted molecular and mAb-based therapies for MCC are promising strategies that depend on our evolving understanding of tumour biology and disease pathogenesis.^44^ To this end, we report the first evidence of prevalent FOLH1 expression within MCC-associated neo-vessels, in 60-77% of patients in a large MCC cohort. We also demonstrate the first illustrative step towards dose calculation and radiation treatment planning for antibody-based brachytherapy (patent pending) via Monte Carlo simulation and immunohistochemistry. Given this data, and the need for alternatives to immune therapies, it is appropriate to explore the safety and efficacy of FOLH1-targeted brachytherapy for MCC.

## Figures and Tables

**FIGURE 1:**
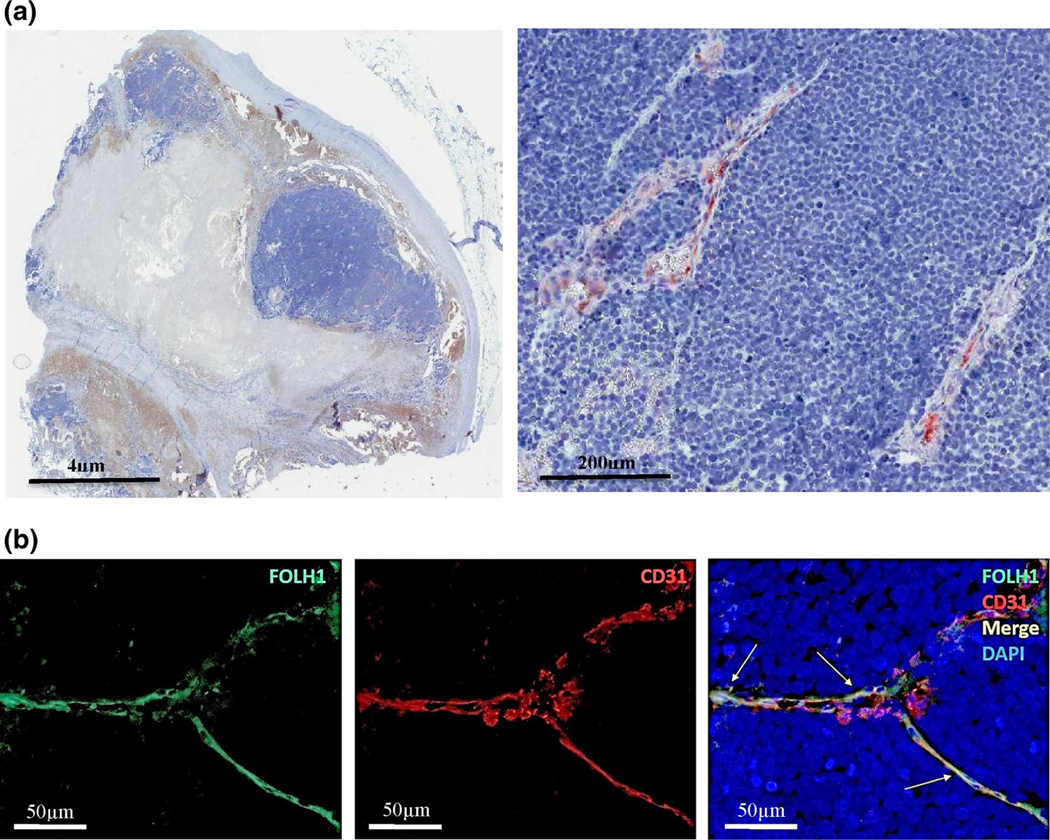
FOLH1 is expressed in the neo-vasculature of primary and meta-stastic Merkel cell carcinoma (a) Immunohistochemistry staining of metastatic MCC paraffin sections with a mouse IgG1 monoclonal anti-human FOLH1. (b) Immunofluorescent co-labelling of FOLH1 (green) with the endothelial marker CD31 (red) in a case of meta-static MCC. Arrows indicate co-labelling of FOLH1 and CD31 (yellow). FOLH1, folate hydrolase-1; MCC, Merkel cell carcinoma

**FIGURE 2:**
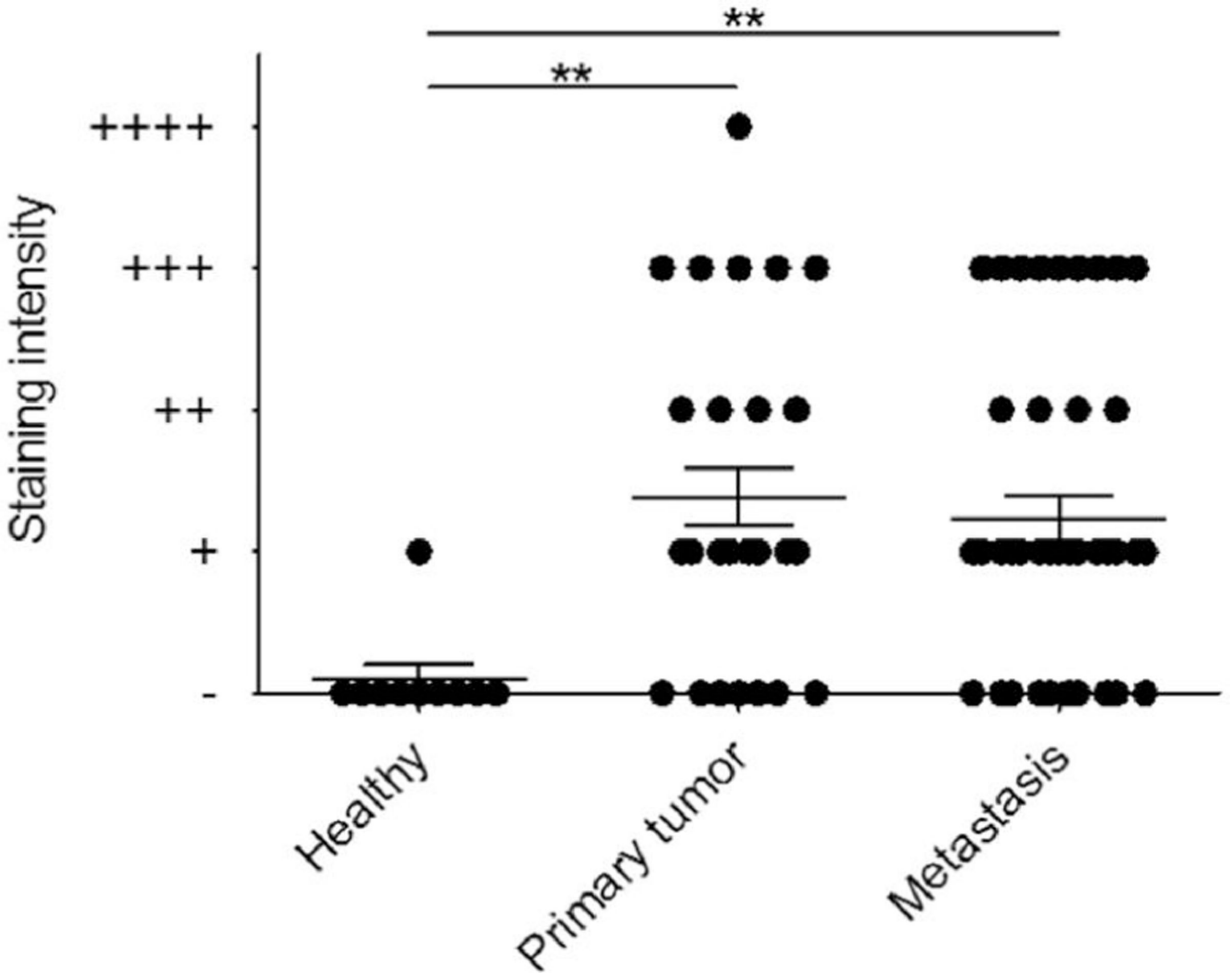
Semi-quantification of FOLH1 staining intensity. The MCC cases were classified as (−), (+), (++), (+++) and (++++) staining intensities; (++++) was characterized as maximal staining as seen in prostate cancer where there is both cellular and neo-vascular staining. Both primary and metastatic MCC expressed significantly more FOLH1 as compared to healthy skin. **p < 0.01. FOLH1, folate hydrolase-1; MCC, Merkel cell carcinoma

**FIGURE 3:**
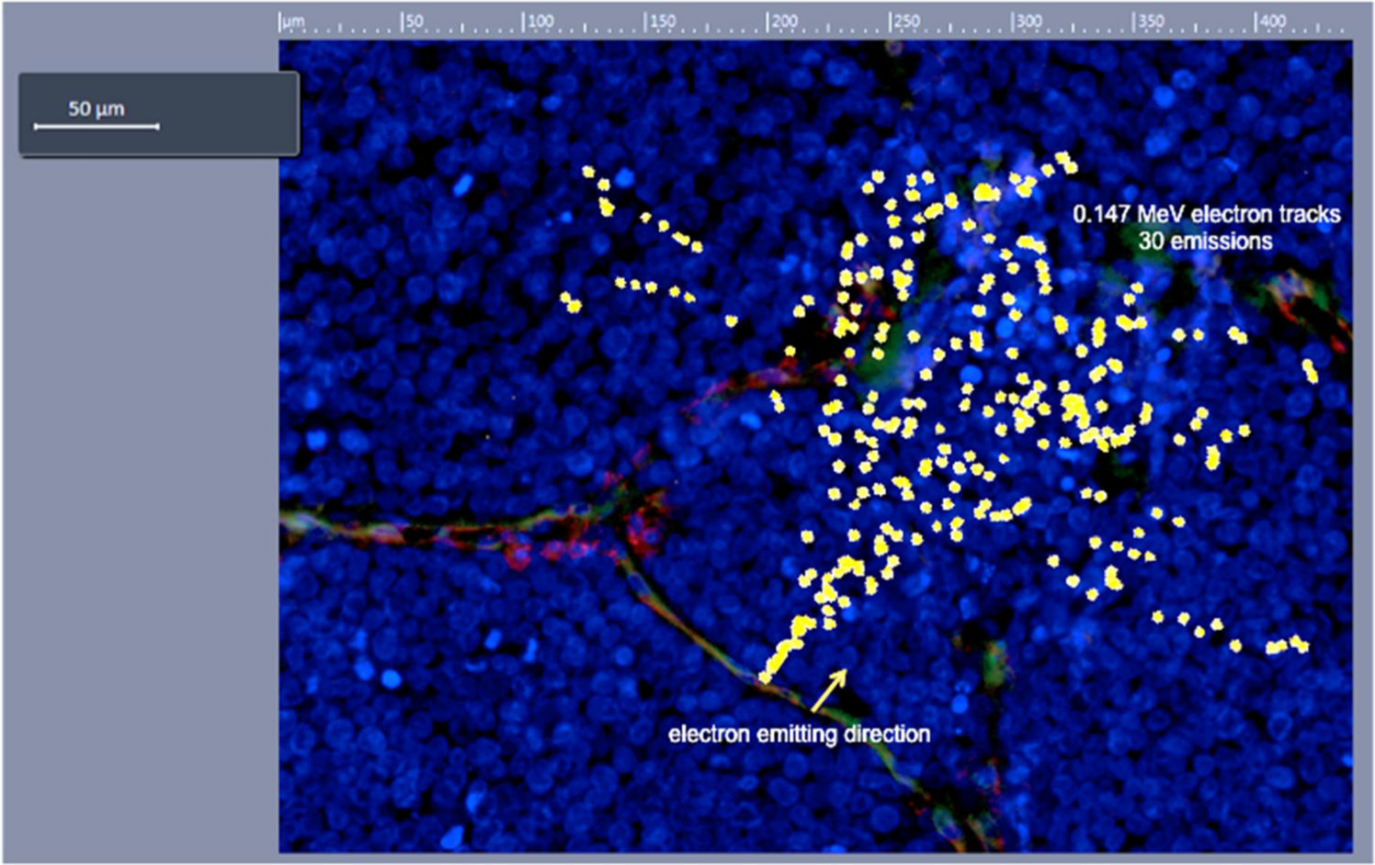
A demonstration of the track structure of 30 electrons emitted from ^177^Lu. The natural decay of ^177^Lu occurs spherically from its point source; for clarity, only electrons emitted in a single direction and visible in a single plane that is perpendicular to neo-vessel blood flow were depicted. A mean energy of 0.147 MeV was chosen to best depict the electron tracks. The figure demonstrates 30 electrons with the same energy of 0.147 MeV emitted from the same point on a neo-vessel within a MCC tumour, in the same 2-D direction. Sites of overlapping ionization points and tumour cell nuclei (DAPI; Blue) are sites of potential DNA damage and subsequent cell death. Note, that the apparent discontinuity of the tracks are due to the fact that electrons in and out of the 2-D plane depicted in this figure, i.e., the tracks scatter above and below the plane). MCC, Merkel cell carcinoma

**FIGURE 4:**
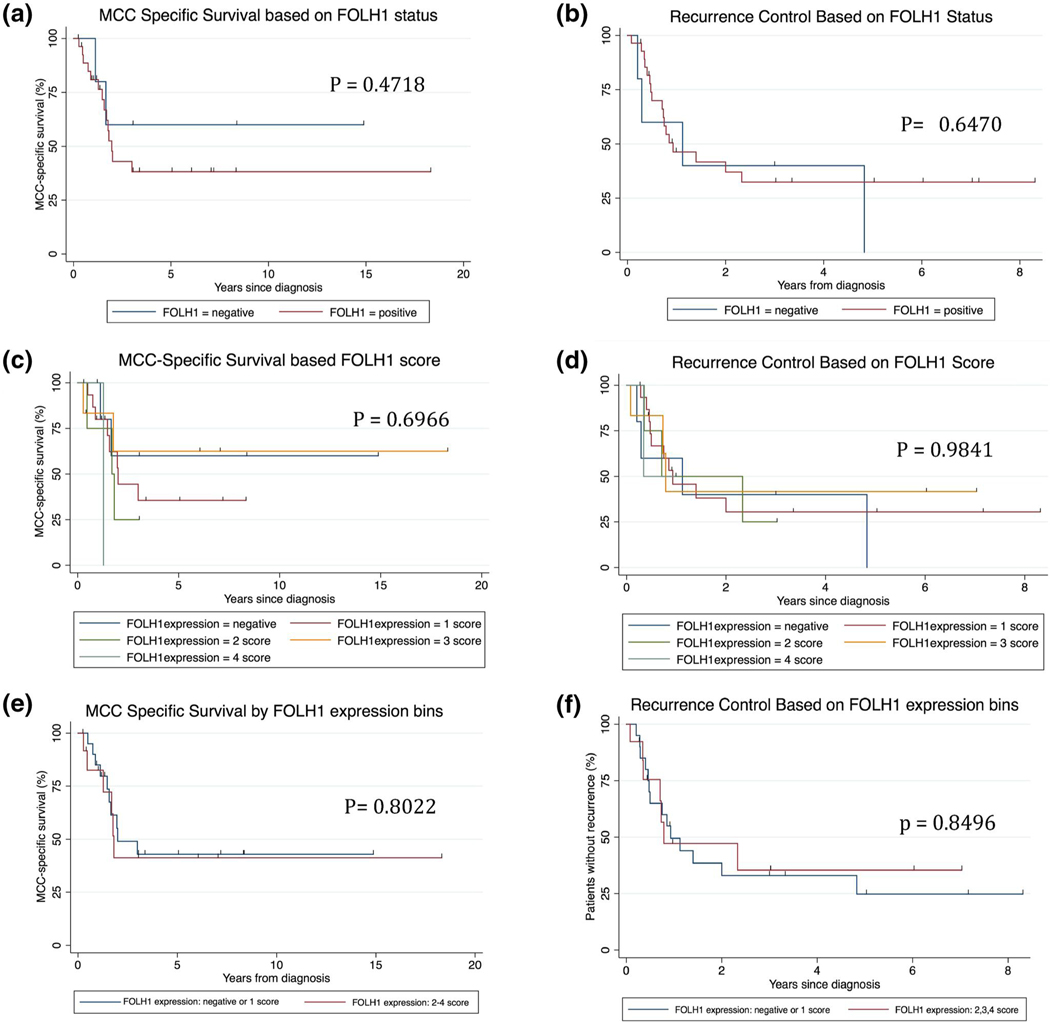
Kaplan–Meier curves for MCC-specific survival and recurrence free survival. No significant differences were detected based on (a and b) FOLH1 status (*p* = 0.4718; *p* = 0.6470), (c and d) staining intensity score (*p* = 0.6966; *p* = 0.9841), or by (e and f) grouping staining intensity scores (− and + vs. ++, +++, +++) (*p* = 0.8022; *p* = 0.8496) for MCC specific survival or recurrence free survival, respectively. FOLH1, folate hydrolase-1; MCC, Merkel cell carcinoma

**TABLE 1:** Patient demographics

Characteristic	FOLH1 negative (*n* = 6)	FOLH1 positive (*n* = 29)	Total cohort (*n* = 35) *P*
Sex			
Male (*n* = 21)	3	18	0.664
Female (*n* = 14)	3	11	-
Age at diagnosis			
≥65 (*n* = 26)	3	23	0.162
<65 (*n* = 9)	3	6	-
Immunosuppressed			
Yes (*n* = 5)	0	5	0.539
No (*n* = 18)	4	14	-
Received RT to primary site			
Yes (*n* = 17)	4	13	1
No (*n* = 3)	0	3	-
Stage number			
IA (*n* = 1)	1	0	0.317
IB (*n* = 2)	0	3	-
IIA (*n* = 3)	0	2	-
IIB (*n* = 2)	0	2	-
IIIA (*n* = 7)	2	5	-
IIIB (*n* = 5)	0	5	-
IV (*n* = 3)	1	2	-
Sentinel lymph node biopsy performed?			
Yes (*n* = 13)	3	8	0.616
No (*n* = 9)	1	8	-
Site			
Head & neck (*n* = 14)	4	10	0.605
Trunk (*n* = 2)	0	2	-
Upper limb (*n* = 7)	0	7	-
Lower limb (*n* = 9)	2	7	-
Unknown primary (*n* = 1)	0	1	-
Received chemotherapy			
Yes (*n* = 6)	2	4	0.549
No (*n* = 14)	2	12	-
Local/Regional recurrence			
Yes (*n* = 8)	1	7	1
No (*n* = 14)	3	11	-
Draining LN recurrence			
Yes (*n* = 11)	1	10	1
No (*n* = 9)	2	7	-
Distant metastatic recurrence			
Yes (*n* = 13)	2	11	0.587
No (*n* = 7)	2	5	-
MCC cells			
50%–75% (*n* = 7)	2	5	0.587
76%–100% (*n* = 13)	2	11	-

Abbreviations: FOLH1, folate hydrolase-1; LN, lymph node; MCC, Merkel cell carcinoma; RT, radiotherapy.

## What’s already known about this topic?

- We report the first evidence of prevalent folate hydrolase-1 (FOLH1; also known as prostate-specific membrane antigen) expression within MCC-associated neovessels.

## What does this study add?

- Herein, FOLH1 expression in Merkel cell carcinoma neovasculature is validated, and the therapeutic mechanism of specific, systemic targeting of disseminated disease with antibody-based brachytherapy, is defined.
